# Novel B-Cell targeting therapy with subcutaneous ofatumumab in AQP4-IgG-seronegative Neuromyelitis Optica Spectrum Disorders: efficacy and personalized dosing

**DOI:** 10.3389/fimmu.2026.1760491

**Published:** 2026-03-13

**Authors:** Xuetao Cao, Danqing Qin, Baojie Wang, Chunjuan Wang, Shougang Guo

**Affiliations:** 1 Department of Neurology, Shandong Provincial Hospital Affiliated to Shandong First Medical University, Jinan, Shandong, China; 2 Department of Neurology, Shandong Provincial Hospital, Shandong University, Jinan, Shandong, China; 3 Department of Geriatric Neurology, Shandong Provincial Hospital Affiliated to Shandong First Medical University, Jinan, Shandong, China

**Keywords:** AQP4-IgG-seronegative NMOSD, central nervous system demyelinating disease, novel B-cell targeting therapy, ofatumumab, personalized medicine

## Abstract

**Background and objectives:**

AQP4-IgG-seronegative Neuromyelitis Optica Spectrum Disorders (AQP4-IgG-seronegative NMOSD) represent a distinct and rare subtype of Neuromyelitis Optica Spectrum Disorders (NMOSD). Diagnosis and management of this condition pose significant challenges in clinical practice. Here, we present two cases of AQP4-IgG-seronegative NMOSD, which demonstrated a favorable response to personalized ofatumumab (OFA) therapy.

**Methods:**

Two patients, confirmed negative for both AQP4-IgG and MOG-IgG by cell-based assay methods and meeting the diagnostic criteria for AQP4-IgG-negative NMOSD according to the 2015 international criteria were treated with monthly subcutaneous OFA (20 mg). Clinical status was monitored using the Expanded Disability Status Scale (EDSS), B-cell depletion (CD19+%), MRI, and serum neurofilament light chain (NfL).

**Results:**

Both patients (a 13-year-old male and a 31-year-old female) had severe disability (EDSS 6.5 and 5.5, respectively) and poor response to initial steroids/IVIG. After OFA initiation, both achieved rapid and sustained B-cell depletion (CD19+% 0.00-0.11%). Symptoms remained stable and gradually improved, imaging showing marked reduction or resolution of lesions and EDSS scores decreasing by ≥3 points (to 2.0 in both patients) over 18–20 months of follow-up. No clinical relapses or serious adverse events (e.g., infections, significant IgG reduction) occurred. After 12 months of monthly dosing, the interval was successfully extended to every two months in both patients while maintaining efficacy and B-cell depletion.

**Discussion:**

Subcutaneous OFA demonstrated sustained efficacy and a favorable safety profile as a long-term therapy for AQP4-IgG-seronegative NMOSD in this case series. It facilitated significant functional recovery, prevented relapses, and enabled personalized, extended dosing intervals. These preliminary findings support OFA as a promising, convenient therapeutic option for AQP4-IgG-seronegative NMOSD, meriting further investigation in larger prospective studies.

## 1 Introduction

Neuromyelitis Optica Spectrum Disorder (NMOSD) is a rare autoimmune disorder of the central nervous system (CNS) that predominantly affects the optic nerve and spinal cord (1). Although NMOSD is typically associated with either Aquaporin-4 (AQP4) or myelin oligodendrocyte glycoprotein IgG (MOG-IgG), a subset of patients remain double seronegative and lack definitive diagnostic markers (2, 3). According to current diagnostic criteria (1), MOG-IgG antibody-associated disease (MOGAD) has been recognized as a distinct clinical entity (4). Accordingly, the patients in this study, who tested seronegative for both AQP4-IgG and MOG-IgG, were classified as having AQP4-IgG-negative NMOSD. These individuals may ultimately have other inflammatory CNS disorders. Therefore, AQP4-IgG-seronegative NMOSD is not a single disease entity but rather a syndrome with diverse therapeutic requirements. Although AQP4-IgG-seronegative NMOSD may follow a monophasic or relapsing course compared to AQP4-IgG-positive NMOSD, it can result in disability as severe as that in AQP4-IgG-positive NMOSD (5), and some patients exhibit a poor response to acute-phase therapy. Therefore, identifying highly effective treatments and preventing disease relapse are crucial to preserve patients’ quality of life.

Currently, B-cell-targeted therapies, such as Rituximab (RTX) (6), Ofatumumab (OFA) (7), Inebilizumab (8), ocrelizumab (9) and ublituximab (10) have demonstrated significant efficacy in treating demyelinating diseases of the central nervous system. However, none of agents are yet approved for use in AQP4-IgG-seronegative NMOSD. Ofatumumab, a fully human, next-generation anti-CD20 monoclonal antibody (11), demonstrates stronger complement-dependent cytotoxicity *in vitro* and lower immunogenicity (12, 13). Crucially, for some patients, once-monthly subcutaneous administration may offer a more flexible dosing option. However, despite its mechanistic advantages, published evidence specifically on OFA in AQP4-IgG-seronegative NMOSD remains scarce, with only limited data available from studies that included a small number of seronegative patients (14). To address this gap, we present two cases with complex clinical courses, ultimately diagnosed with AQP4-IgG-seronegative NMOSD, who were successfully treated with subcutaneous OFA, and further explore the feasibility of personalized dosing extension. The studies involving human participants were reviewed and approved by the Ethics Institutional Review Board of Shandong Provincial Hospital. Written informed consent to participate in this study was provided by the participants or their legal guardians/next of kin. Written informed consent was obtained for the publication of any potentially identifiable images or data included in this article.

## 2 Case presentation

### 2.1 Case 1

**Figure 1:**
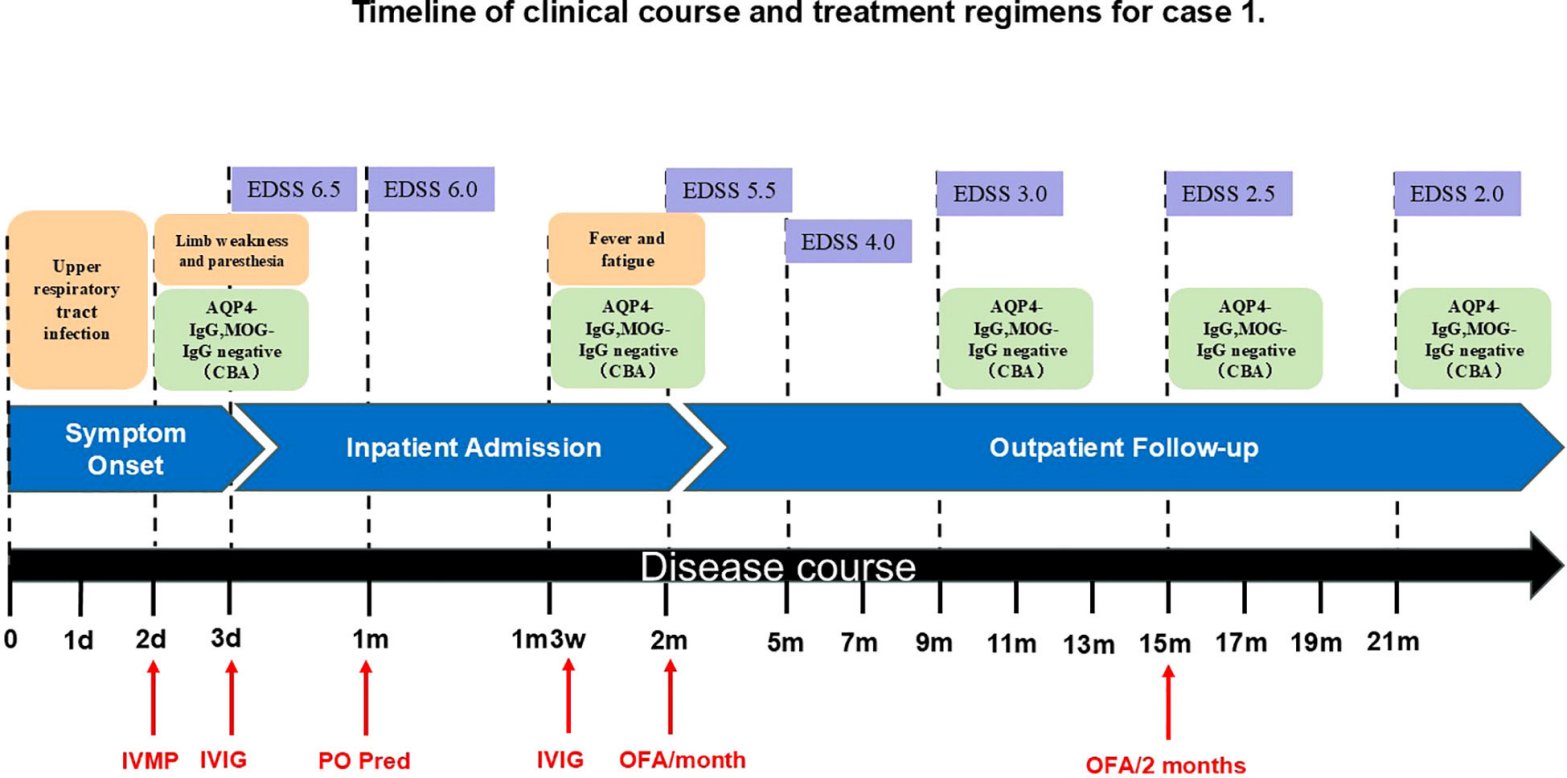
Timeline of clinical course and treatment regimens for case 1. AQP4-IgG, Aquaporin-4 Immunoglobulin G; CBA, Cell-Based Assay; d, day/days; EDSS, Expanded Disability Status Scale; IVIG, Intravenous Immunoglobulin; IVMP, Intravenous Methylprednisolone; m, month/months; MOG-IgG, Myelin Oligodendrocyte Glycoprotein Immunoglobulin G; OFA, Ofatumumab; PO Pred, Per Os Prednisone.

**Figure 2:**
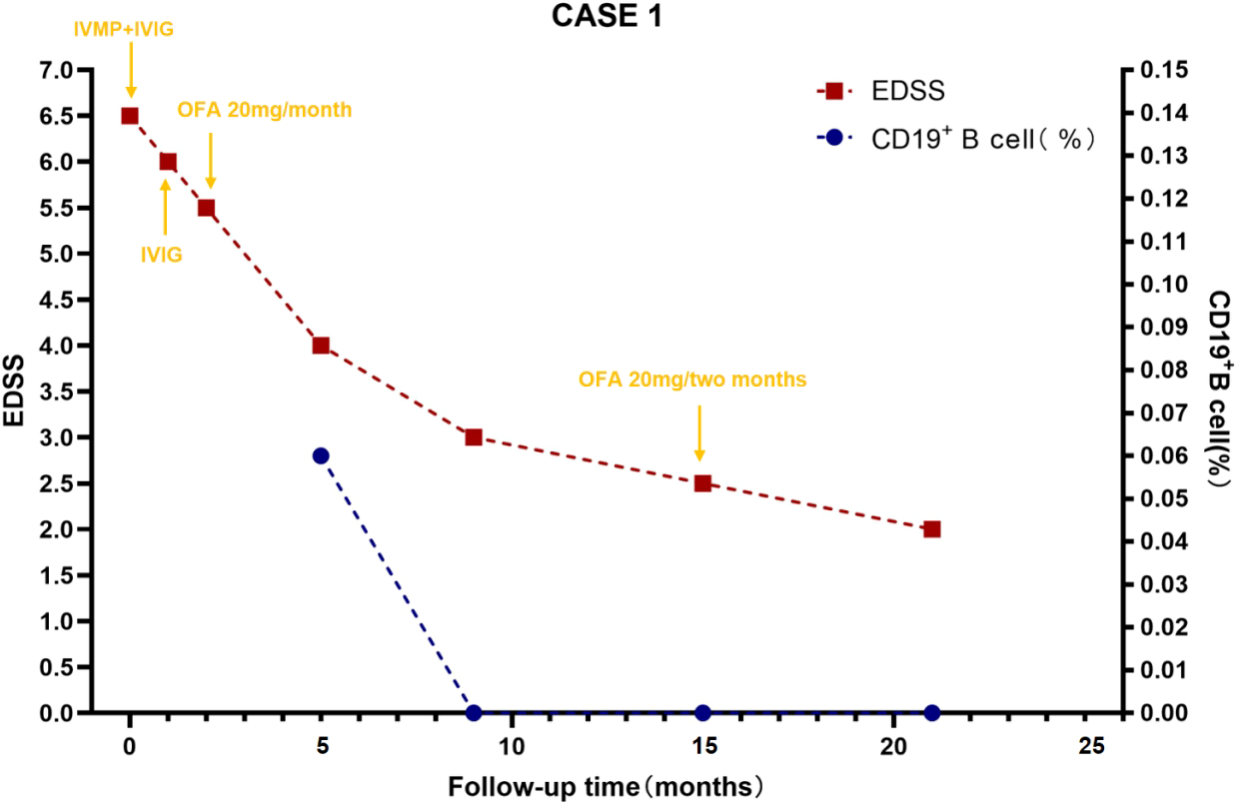
B-cell levels and expanded disability status scale scores during ofatumumab treatment for case 1. B-cell levels were measured via flow cytometry. CD19+B cell (%) reflects the percentage of total B lymphocytes expressing CD19+ among lymphocytes, which is used to evaluate the efficacy of immunotargeted therapy. EDSS, Expanded Disability Status Scale; IVIG, Intravenous Immunoglobulin; IVMP, Intravenous Methylprednisolone; OFA, Ofatumumab.

**Figure 3:**
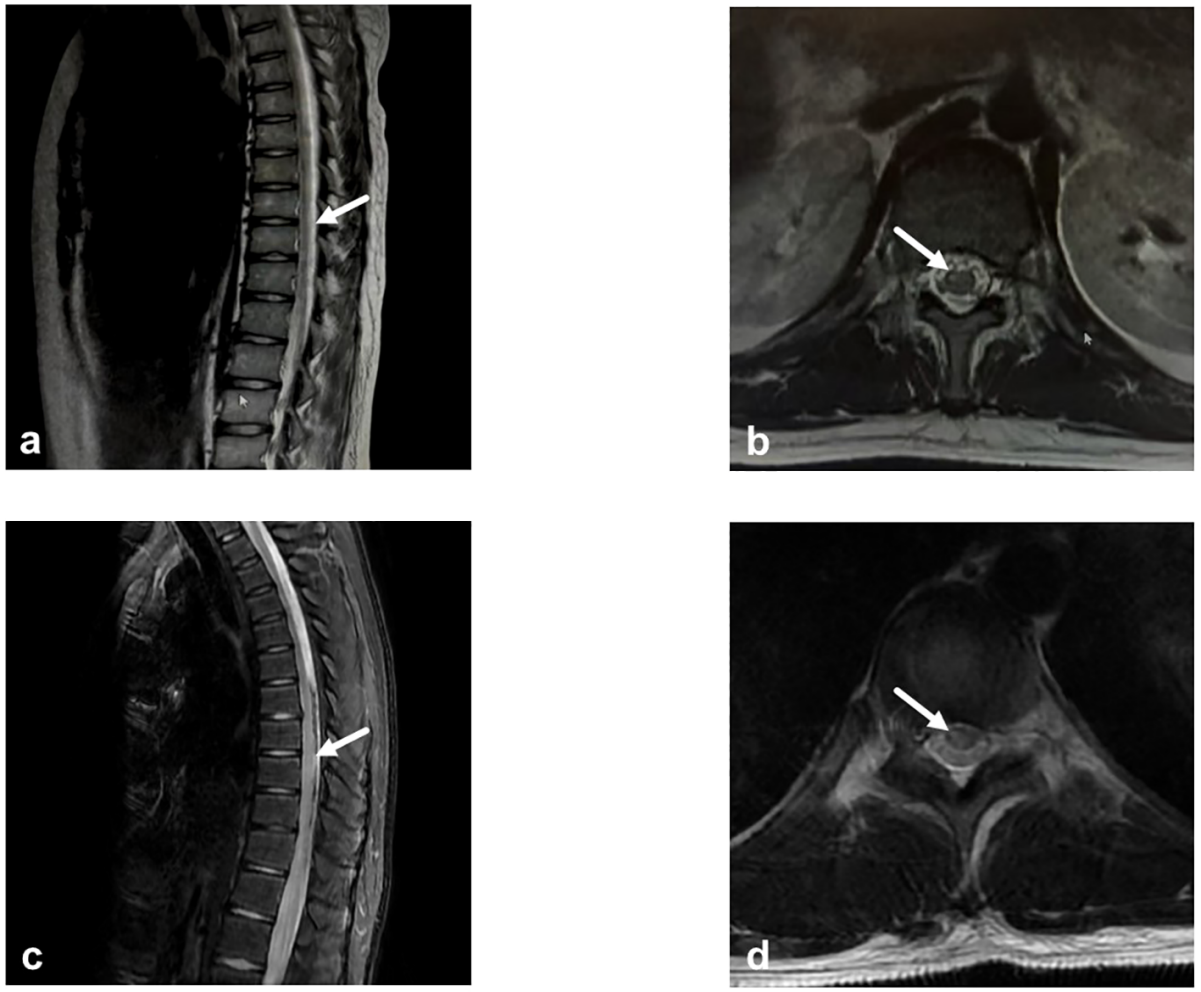
Imaging changes before and after ofatumumab treatment for case 1. **(a)** Sagittal T2-weighted thoracic spinal MRI. A patchy area of T2-weighted hyperintensity is observed within the spinal cord from T6 to L1, consistent with myelitis. There is a slight increase in epidural fat within the lumbar and sacral canal, resulting in compression of the thecal sac. **(b)** Axial T2-FLAIR thoracic spinal MRI. Reveals an abnormal signal focus in the right side of the spinal cord. **(c)** Sagittal T2-weighted thoracic spinal MRI. Demonstrates post-myelitis treatment changes. Following OFA treatment, thoracic spinal cord imaging shows a significant reduction in the extent of diffuse signal intensity compared to previous studies. **(d)** Axial T2-FLAIR thoracic spinal MRI. The previously noted abnormal signal in the right spinal cord has nearly resolved. OFA, Ofatumumab.

In October 2023, a 13-year-old boy was admitted to a local hospital with progressive weakness and sensory abnormalities in both lower limbs for 2 days, accompanied by unsteady gait. Notably, the patient had a common cold two days before symptom onset. He received pulse methylprednisolone therapy but responded poorly during tapering, his symptoms showed no improvement and even worsened further. Then he was transferred to our hospital. Physical examination on admission showed muscle strength of grade 1 in both lower limbs, sensory impairment below the umbilicus, absent tendon reflexes in the lower limbs, and positive bilateral Babinski signs. The Expanded Disability Status Scale (EDSS) score was 6.5, indicating severely restricted walking ability without assistance. Repeat spinal MRI revealed longitudinally extensive transverse myelitis lesions spanning the T6 to L1 segments, which appeared hyperintense on T2-weighted imaging and mildly hypointense on T1-weighted imaging in some areas. Contrast-enhanced scans showed scattered punctate enhancement within the lesions. Optic nerve MRI was unremarkable. On day 6, lumbar puncture was performed, revealing elevated cerebrospinal fluid (CSF) protein (0.48 g/L) and a white blood cell count of 10×10^6^/L (all mononuclear). Comprehensive screening for infections (including HIV antigen/antibody, syphilis antibody, Lyme disease antibody, CMV DNA, EBV DNA, HSV DNA, and VZV DNA), metabolic disorders (homocysteine, folate, and vitamin B12 levels), and systemic autoimmune diseases (ANA, anti-Sm, anti-dsDNA, anti-SSA, anti-SSB antibodies, thyroglobulin, and thyroid peroxidase antibodies) were all negative. Chest CT showed no mediastinal lymphadenopathy, and serum ACE levels were normal, further ruling out the possibility of sarcoidosis. Serum and CSF tests for AQP4-IgG, GFAP-IgG, MBP-IgG, MOG-IgG (live cell-based assay, CBA), and oligoclonal bands (OCBs) were negative. Antibodies for autoimmune encephalitis (including anti-NMDAR, anti-LGI1, anti-GABA, anti-mGluR5, etc.) detected by CBA and Tissue-based Assay (TBA), paraneoplastic antibodies (anti-Hu, Yo, Ri, SOX1, GAD65, etc. CBA+TBA), and peripheral neuropathy antibodies (anti-GQ1b, GD1b, GM1, etc.) were also negative. Initial treatment with one course of intravenous immunoglobulin (IVIG) and pulse methylprednisolone led to partial improvement, though the EDSS score decreased only to 6.0, with persistent walking limitations. Follow-up MRI showed no significant change in the lesions. He was discharged with a diagnosis of myelitis and maintained on oral prednisone (60 mg/day). Three weeks later, he returned with generalized fatigue and unresolved weakness in the lower limbs. Upon readmission, muscle strength was grade 4 in the left lower limb, grade 3 distally and grade 4 proximally in the right lower limb, with bilateral Babinski signs. A throat swab was positive for influenza A virus. The total B-cell/lymphocyte ratio was elevated at 33.29%. Repeat tests for OCBs, AQP4-IgG, and MOG-IgG remained negative. Serum neurofilament light chain (NfL) was elevated at 11.87 pg/mL (reference <40 years: 0.00–8.10 pg/mL), suggesting ongoing neuroaxonal injury. Based on the presence of longitudinally extensive spinal cord lesions, negative serum and CSF OCBs, and suboptimal response to acute-phase treatment, the patients did not meet the diagnostic criteria of 2017 for multiple sclerosis (MS) (18). Based on the 2015 international criteria, he was diagnosed with AQP4-IgG-seronegative NMOSD. Besides antiviral treatment for influenza, another course of IVIG was administered. Follow-up MRI before discharge showed slightly reduced signal intensity in the spinal lesions, and the EDSS score improved to 5.5. Considering the slow onset of conventional immunosuppressants and potential long-term toxicity in pediatric patients, Given the established evidence supporting favorable outcomes with B-cell targeted therapies in various CNS demyelinating diseases, we opted for this approach to alleviate symptoms and prevent relapse. OFA was ultimately selected as it offers greater convenience and a more favorable safety profile compared to RTX. After excluding contraindications and obtaining informed consent, subcutaneous ofatumumab (OFA, 20 mg/month) was initiated. During the 12-month period following medication initiation, the patient progressed from being unable to walk independently to regaining unrestricted walking ability. The EDSS score decreased to 2.5, while the CD19+ B-cell percentage (CD19+%) remained consistently low or even reached undetectable levels. Subsequently, based on economic considerations, the stability of the patient’s symptoms, and the sustained stable depletion of B cells, the OFA dosing interval was extended to once every two months. By the 18-month follow-up, the total B-cell/lymphocyte count was still maintained at 0.00%, and the EDSS score had stabilized at 2.0. Changes in patient symptoms, EDSS scores, and B-cell ratios are shown in **Figures 1**, **2**. Imaging changes following treatment are presented in **Figure 3**. Serial tests for AQP4-IgG and MOG-IgG remained negative and serum NfL levels decreased to 3.63 pg/mL at the most recent follow-up. Notably, no serious adverse events, including infections, were observed, and no significant reduction in serum immunoglobulin IgG levels was detected in the patients.

### 2.2 Case 2

**Figure 4:**
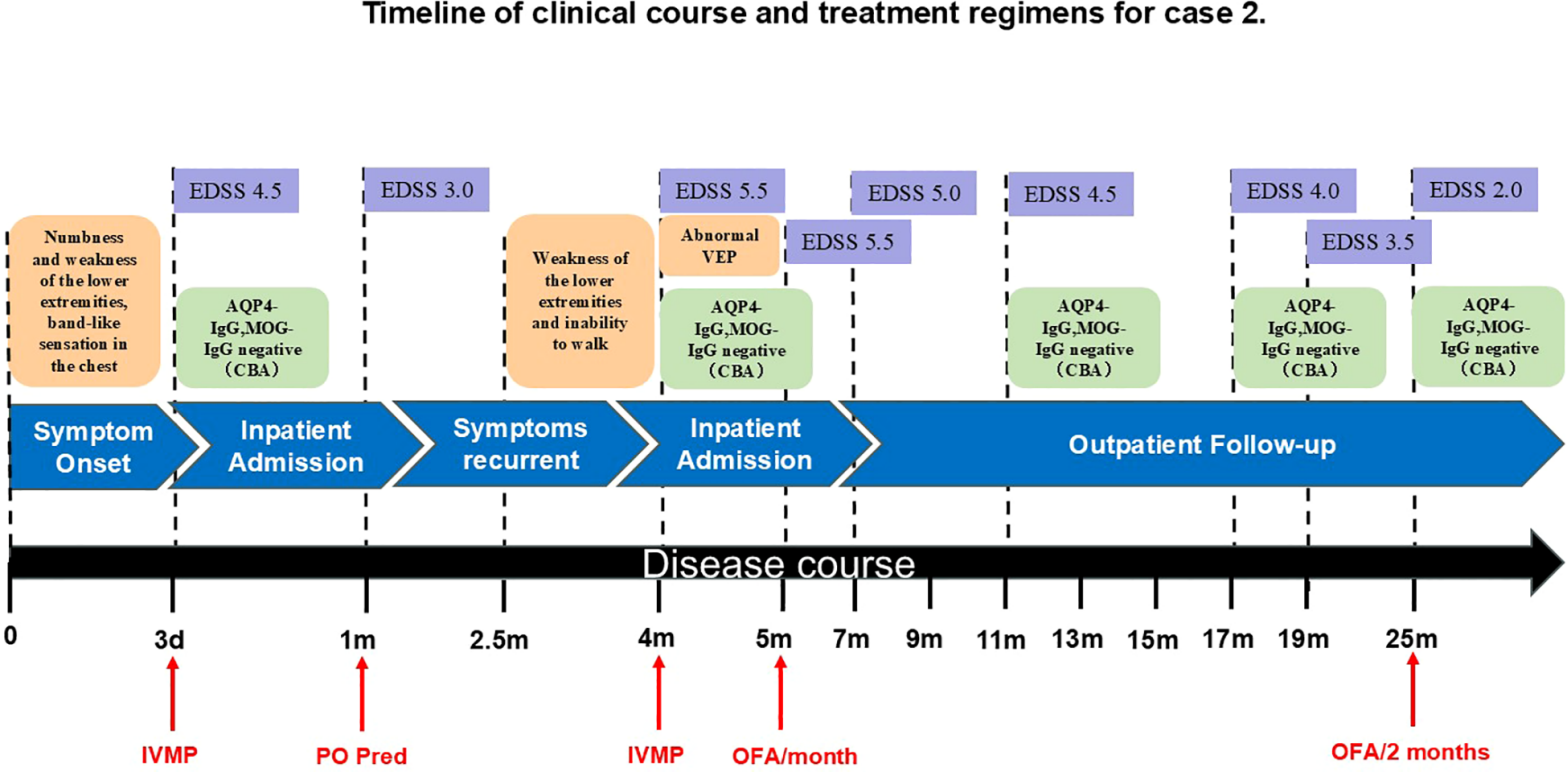
Timeline of clinical course and treatment regimens for case 2. AQP4-IgG, Aquaporin-4 Immunoglobulin G; CBA, Cell-Based Assay; d, day/days; EDSS, Expanded Disability Status Scale; IVMP, Intravenous Methylprednisolone; m, month/months; MOG-IgG, Myelin Oligodendrocyte Glycoprotein Immunoglobulin G; OFA, Ofatumumab; PO Pred, Per Os Prednisone; VEP, Visual Evoked Potential.

**Figure 5:**
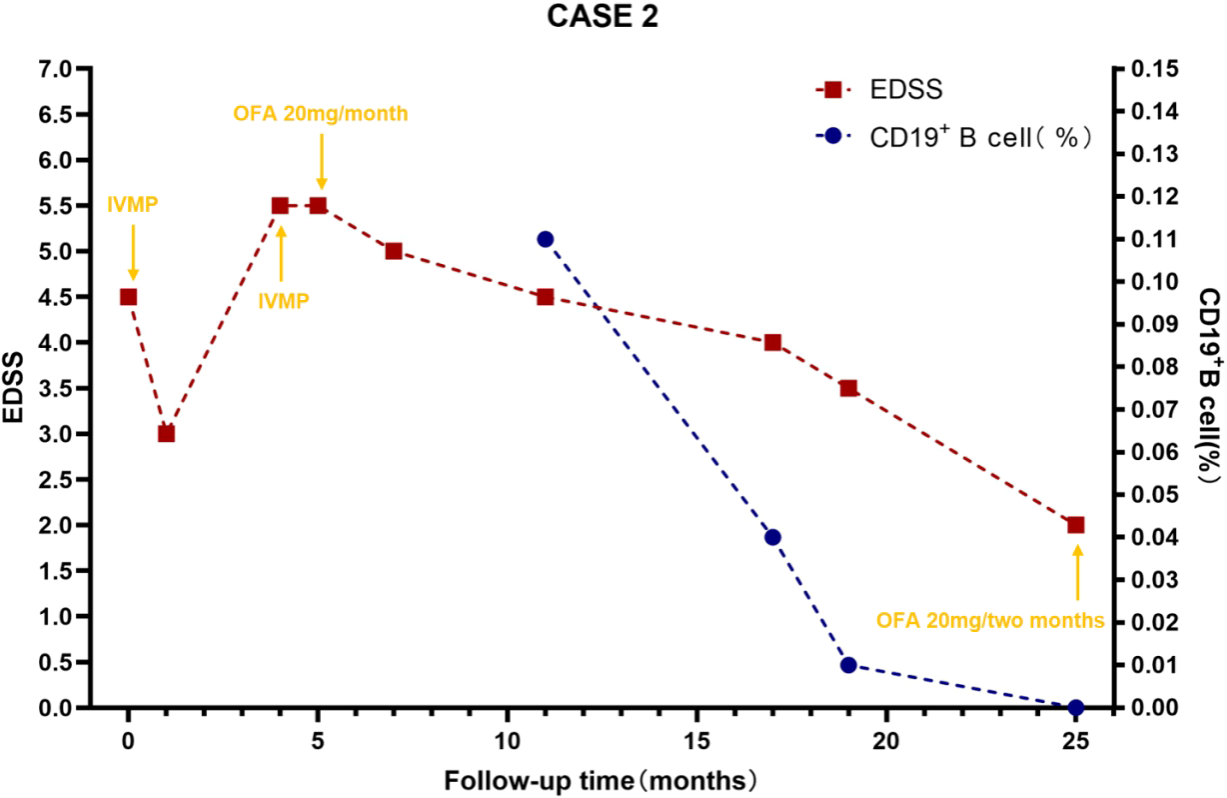
B-cell levels and expanded disability status scale scores during ofatumumab treatment for case 2. B-cell levels were measured via flow cytometry. CD19+B cell (%) reflects the percentage of total B lymphocytes expressing CD19+ among lymphocytes, which is used to evaluate the efficacy of immunotargeted therapy. EDSS, Expanded Disability Status Scale; IVMP, Intravenous Methylprednisolone; OFA, Ofatumumab.

**Figure 6:**
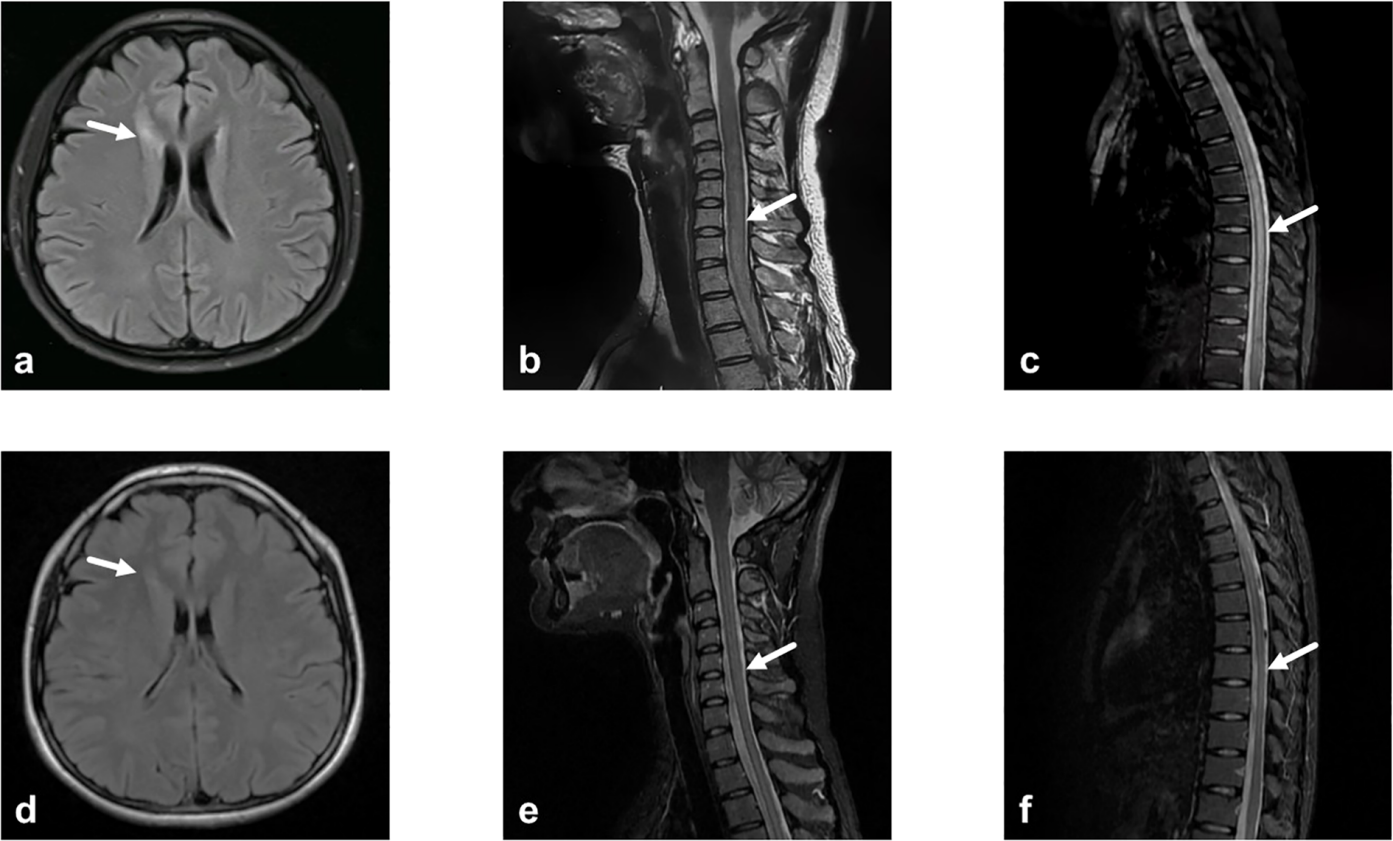
Imaging changes before and after ofatumumab treatment for case 2. **(a)** Axial T2-FLAIR brain MRI. A patchy FLAIR hyperintensity lesion is observed in the white matter adjacent to the right frontal horn of the lateral ventricle. **(b)** Sagittal T2-weighted cervical spinal MRI. The lower cervical spinal cord is swollen with diffuse T2-weighted hyperintensity, consistent with myelitis of the lower cervical cord. Long-segment hyperintensity spanning ≥3 vertebral segments is consistent with LETM. **(c)** Sagittal T2-weighted thoracic spinal MRI. The thoracic spinal cord is swollen with diffuse T2-weighted hyperintensity, consistent with thoracic myelitis. Long-segment hyperintensity spanning ≥3 vertebral segments is observed. **(d)** Axial T2-FLAIR brain MRI. Following OFA treatment, the lesion in the white matter adjacent to the right frontal horn of the lateral ventricle has completely resolved. **(e)** Sagittal T2-weighted cervical spinal MRI. After OFA treatment, a patchy T2-weighted hyperintensity is visible within the cervical spinal cord, showing significant reduction in extent and marked improvement of edema compared to previous imaging. **(f)** Sagittal T2-weighted thoracic spinal MRI. After OFA treatment, a patchy T2-weighted hyperintensity is visible within the thoracic spinal cord, showing significant reduction in extent and improvement of edema compared to previous imaging. FLAIR, fluid-attenuated inversion recovery; LETM, longitudinal extensive transverse myelitis; OFA, Ofatumumab.

A 31-year-old woman presented to an external hospital in June 2023 with numbness and weakness in both lower limbs, unsteady gait when walking uphill, and a band-like sensation around the chest. Electromyography indicated bilateral pyramidal tract damage. Cranial, cervical, and thoracolumbar MRI revealed long-segment abnormal signals in the lower cervical and thoracic cord (C5–T2). Serum and CSF tests for AQP4-IgG, GFAP-IgG, MBP-IgG, MOG-IgG (CBA) and OCBs were negative. She received pulse methylprednisolone. Follow-up MRI showed improvement in myelitis, and her EDSS score decreased from 4.5 to 3.0. She was discharged with a diagnosis of myelitis and maintained on oral prednisone (60 mg/day). Unfortunately, 2.5 months later, while tapering to 45 mg/day, she experienced recurrence of lower limb weakness, inability to walk independently, and progressive worsening. She was referred to our hospital. Physical examination revealed muscle strength of grade 4+ in the left lower limb and grade 4 in the right lower limb, increased muscle tone in the right lower limb, hyperactive tendon reflexes in both lower limbs, positive bilateral babinski signs, positive left Hoffmann sign, and impaired superficial sensation below the nipple line bilaterally. The EDSS score worsened to 5.5. Repeat MRI showed diffuse intramedullary abnormal signals in the spinal cord from C4-T7, with central edema and enhancement. CSF analysis revealed a white blood cell count of 1 cell/μL, predominantly lymphocytes, with elevated protein levels (0.922 g/L) and negative OCBs. She received another course of pulse methylprednisolone but responded poorly. Although alternative first-line treatment strategies needed to be considered, IVIG was not initiated as her symptoms had worsened over 20 days prior to her referral to our hospital. During treatment, visual evoked potential (VEP) testing and repeat MRI of the brain and optic nerves were performed. Although the patient remained asymptomatic, the VEP revealed left-sided abnormalities, and MRI demonstrated a lesion near the right frontal horn, and left optic neuritis (ON)—indicating potential subclinical optic nerve injury. Repeat tests for AQP4-IgG, GFAP-IgG, MBP-IgG, MOG-IgG remained negative. Additional tests ruled out metabolic causes (including serum vitamin B12, ceruloplasmin, thyroid function, etc.), infectious causes (including HIV antigen/antibody, syphilis antibody, Lyme disease antibody, CMV DNA, EBV DNA, HSV DNA, and VZV DNA), neoplastic causes (tumor marker screening, whole-body PET-CT), and vascular causes (ANCA, spinal cord CTA). Paraneoplastic antibodies (CBA+TBA) were negative. Serum NfL was elevated at 19.38 pg/mL. A final diagnosis of AQP4-IgG-seronegative NMOSD was made. Due to early relapse, Similarly to Case 1, we selected a maintenance therapy regimen consisting of oral prednisone and subcutaneous OFA monthly. By 6 months, she could walk independently with limited distance. The CD19+ B-cell percentage (CD19+%) was 0.11%, and the EDSS score was 4.5. At 12 months, walking was largely unrestricted (EDSS 4.0, CD19+ B-cell percentage 0.04%). At the latest follow-up (20 months), superficial sensory impairment was confined to the right foot with mildly increased muscle tone. The CD19+ B-cell percentage was 0.00%, and the EDSS score improved to 2.0. Subsequently, OFA was adjusted to once every two months. Changes in patient symptoms, EDSS scores, and B-cell ratios are shown in **Figures 4**, **5**. Imaging changes following treatment are presented in **Figure 6**. Repeated tests for AQP4-IgG and MOG-IgG remained negative and the patient’s serum NfL level decreased to 5.54 pg/mL at the final follow-up. No infections or reduction in immunoglobulin IgG levels were observed throughout the treatment course.

## 3 Discussion

To our knowledge, this case series represents the first report on the application of subcutaneous OFA in the treatment of AQP4-IgG-seronegative NMOSD, with exploration of personalized dosing. We demonstrate that subcutaneous OFA, induced rapid and sustained B-cell depletion, improved EDSS scores from baseline, radiologic evidence of lesion resolution, and complete relapse prevention over 18–20 months in two severely disabled AQP4-IgG-seronegative NMOSD patients. A key finding of our report is the successful extension of the dosing interval from monthly to every two months while maintaining efficacy and B-cell depletion. This offers a promising strategy for treatment personalization, which could reduce the treatment burden and cost for patients. These two cases of AQP4-IgG-seronegative NMOSD highlight several important clinical and therapeutic considerations. Continued regular follow-up is planned to monitor their improvement, particularly after extending the intervals between OFA administrations.

In these two cases, the initial diagnosis was idiopathic acute transverse myelitis (iATM). The AQP4-IgG-seronegative NMOSD diagnosis is particularly challenging because some patients present with a monophasic disease course, isolated lesions, and negative antibody status, making it easily confused with iATM (1). A definitive diagnosis of AQP4-IgG-seronegative NMOSD requires the exclusion of various other causes, alongside highly sensitive cell-based assays for both MOG-IgG and AQP4-IgG, which poses significant difficulties in clinical practice (1). In terms of clinical characteristics, both patients had LETM lesions and severe disability. AQP4-IgG-seronegative NMOSD is similar to AQP4-IgG-positive NMOSD, leads to severe disability and visual impairment (5, 19–22). However, the average length of LETM lesions in AQP4-IgG-seronegative NMOSD is shorter (19), and it is associated with a lower relapse rate (19, 20, 23). AQP4-IgG-seronegative NMOSD shares the clinical feature of simultaneous ON and TM with MOGAD, but differs by a higher likelihood of LETM and a tendency for lesions to affect the cervical or thoracic cord, rather than the conus medullaris (5, 19–22, 24, 25). Notably, our cases found some patients may lack clinical symptoms of ON, yet investigations reveal VEP abnormalities and ON lesions on MRI. Compared to MS, besides distinct brain lesion patterns (1),unmatched CSF OCBs are found in 8–23% of AQP4-IgG-seronegative NMOSD cases, a rate significantly lower than in MS (19, 26). Both of our patients underwent extensive evaluations, including comprehensive antibody testing, tumor screening, as well as vascular, infectious, and metabolic workups. Under current diagnostic technologies, their conditions met the 2015 diagnostic criteria (1), ultimately confirming the diagnosis of AQP4-IgG-seronegative NMOSD.

Currently, due to diagnostic difficulties, evidence for treating AQP4-IgG-seronegative NMOSD is very limited. Acute attacks are most often managed with conventional therapies used for AQP4-IgG-positive NMOSD and MOGAD, and long-term immunosuppression is employed to prevent relapse (5). However, Consistent with previous studies, patients with AQP4-IgG-seronegative NMOSD may exhibit a poorer response to corticosteroid therapy during relapses (24, 27–29). Presently, no relapse-preventive agents are approved specifically for AQP4-IgG-seronegative NMOSD, therefore, conventional immunosuppressants are used empirically (30). For some patients with severe symptoms, early relapse (<6 months), or younger age, the potential hepatorenal toxicity associated with long-term traditional immunosuppressants is a significant concern, necessitating more efficient and rapid relapse prevention therapies (31).

Compared to iATM, prodromal infections are more common in AQP4-IgG-seronegative NMOSD, and the role of infection as an “immune trigger” is more pronounced, often precipitating disease onset or relapse weeks to months post-infection (32). Although AQP4/MOG antibodies are absent, it is likely an autoimmune disorder mediated by other unidentified autoantibodies, T-cell abnormalities, or complement activation. AQP4-IgG-seronegative NMOSD is less frequently associated with other concurrent autoimmune diseases compared to AQP4-IgG-positive NMOSD, but some patients may still present with autoantibodies such as ANA or anti-SSA (19, 21), further suggesting an underlying autoimmune background. A cohort study showed that CSF levels of glial fibrillary acidic protein (GFAP) and glutamine synthetase (GS) were higher or similar in AQP4-IgG-seronegative NMOSD compared to AQP4-IgG-positive NMOSD, and significantly elevated relative to MOGAD and MS (33). Interleukin-6 (IL-6) levels are also higher (34), and terminal complement complex (TCC) and iC3b are significantly elevated compared to MS and controls (35). NFL levels are markedly higher in AQP4-IgG-seronegative NMOSD than in AQP4-IgG-positive NMOSD or MOGAD (36). Moreover, a significant elevation of serum NFL was also observed in our two patients. Collectively, these findings indicate robust humoral immune activation along with inflammatory astrocytic and neuroaxonal damage in AQP4-IgG-seronegative NMOSD (5), suggesting that AQP4-IgG-seronegative NMOSD may not be an independent disease entity but rather a heterogeneous group driven by unknown antibody- or T-cell-mediated mechanisms (5). Mechanistically, as B cells serve as central regulators of the immune response, targeted B-cell depletion can reduce pathogenic antibody production, mitigate humoral immune-mediated injury, and interrupt key pathogenic pathways, combined with the substantial body of evidence supporting the efficacy of various B-cell-depleting therapies in other CNS demyelinating diseases (8), this provides a strong rationale for anti-CD20 therapy in AQP4-IgG-seronegative NMOSD (15–17, 30, 37).

At the time of treatment decision-making (2023), the targeted drugs approved in China for AQP4-IgG-positive NMOSD included inebilizumab (8) and satralizumab (38). However, medical insurance reimbursement for these drugs was typically strictly limited to seropositive patients, and the costs were extremely high. Case 1 involved a minor male who, on one hand, experienced anxiety toward intravenous infusion and preferred subcutaneous administration. On the other hand, traditional immunosuppressants such as mycophenolate mofetil and azathioprine raised concerns about potential adverse side effects with long-term use. Case 2 involved a woman of childbearing age. Based on the known reproductive toxicity of many traditional immunosuppressants, along with an understanding of the mechanism of B-cell depletion therapy, OFA was ultimately selected as an individualized treatment exploration for both patients. OFA is a fully human, IgG1κ monoclonal antibody that effectively depletes B cells through complement-dependent cytotoxicity (CDC) and antibody-dependent cell-mediated cytotoxicity (ADCC) dual mechanisms (17). Compared to RTX, inebilizumab, ocrelizumab and ublituximab, OFA is administered subcutaneously. This route offers greater convenience and enables a more sustained and gradual depletion of B cells, leading to more stable disease control and preventing B-cell repopulation. This approach prevents the rapid lysis of large numbers of B cells, thereby reducing the release of intracellular contents and minimizing the risk of acute infusion-related reactions (17).

Based on the favorable therapeutic response and profound B-cell depletion (CD19^+^ B cells < 0.01%) observed after 12 months of monthly dosing, we extended the dosing interval to once every two months, tailored to the individual circumstances of each patient. This decision was grounded in the pharmacological and immunological principles of OFA: its mechanism does not rely on maintaining constant serum drug concentrations but rather on achieving sustained clearance of B cells through intermittent dosing (11, 13). Once deep depletion is established, the goal of maintenance therapy shifts to preventing B-cell repopulation, providing a theoretical rationale for interval extension (11–13). We defined an individualized “therapeutic window”—the period during which B-cell reconstitution begins but remains below a threshold likely to trigger relapse—through regular B-cell monitoring (39), thereby exploring a cost-effective dosing strategy while ensuring safety. Although premature interval extension may lead to B-cell reconstitution and increase relapse risk (40), and this association is less clearly defined in seronegative NMOSD than in AQP4-IgG + patients (5, 41), we managed this risk through ongoing clinical and laboratory surveillance. To date, B-cell counts in both patients remain undetectable during the extended interval, with no clinical or radiological relapse. This exploratory approach suggests that treatment de-escalation guided by precise monitoring may be feasible for patients achieving deep remission, potentially reducing treatment burden and improving quality of life, and enabling individualized therapy, though its long-term safety and efficacy require confirmation in larger studies.Clinical practice and the successful extension of the dosing interval in our cases demonstrate that effective remission-phase treatment is crucial for alleviating disability resulting from suboptimal acute-phase therapy and reducing the overall burden of disease. This is particularly important for pediatric patients, for whom treatment options with minimal long-term impact on quality of life are essential. Our study found that following OFA treatment, both patients showed significant symptomatic improvement, with a reduction in EDSS scores of ≥3 points, radiologic evidence of lesion reduction, decrease in serum NfL and no relapse or treatment-related adverse events for over 18 months. These results strongly suggest that OFA is an effective and safe option for treating patients with AQP4-IgG-seronegative NMOSD during the remission phase. Furthermore, in recent years, serum NfL has been established as a key dynamic biomarker reflecting neuroaxonal injury in neuroimmune diseases (42). In NMOSD, studies have confirmed that its levels are significantly correlated with disease activity, severity of attacks, and long-term disability accumulation (43). More importantly, NfL can serve as a sensitive indicator for evaluating treatment response, as effective therapy is often accompanied by a decrease in its levels (44). In the present cases, we observed a declining trend in serum NfL levels following OFA treatment, alongside clinical stabilization. This finding not only provides objective biological evidence of clinical efficacy but also suggests that the treatment may effectively suppress subclinical neuroaxonal damage. In the future, incorporating NfL into routine monitoring could potentially guide more individualized therapeutic decisions, such as assessing the safety of dose reduction or extended dosing intervals (5).

Previously, the study by Yang et al. primarily enrolled AQP4-IgG + patients but also included 8 AQP4-IgG – NMOSD patients, demonstrating clinical and biomarker improvements following OFA treatment (14). In contrast, this article focuses on providing a detailed long-term management report of two AQP4-IgG-negative patients, a population that presents greater diagnostic and therapeutic challenges and involves less understood disease mechanisms. It offers a comprehensive account of the individualized dose-adjustment decision-making process, long-term safety data, and detailed monitoring of B-cell subsets and dynamic serum NfL levels. Our exploratory practice of extending the dosing interval raises new questions regarding the possibility of treatment de-escalation for future research. Therefore, our encouraging findings should be interpreted as preliminary and require validation in larger, prospective cohorts.

## Data Availability

The raw data supporting the conclusions of this article will be made available by the authors, without undue reservation.
